# In the shadow of the welfare society ill-health and symptoms, psychological exposure and lifestyle habits among social security recipients: a national survey study

**DOI:** 10.1186/1751-0759-2-15

**Published:** 2008-09-22

**Authors:** Amir Baigi, Eva-Carin Lindgren, Bengt Starrin, Håkan Bergh

**Affiliations:** 1 General Practice and Public Health, Halland County Council, Falkenberg, Sweden; 2 Department of Public Health and Community Medicine, Göteborg University, Göteborg, Sweden; 3 Department of Social Studies, Karlstad University, Karlstad, Sweden; 4 Faculty of Health and Social Studies, Lillehammer University College, Lillehammer, Norway

## Abstract

**Background:**

In Sweden social security is a means-tested financial allowance. The Social Services Act states that an individual is entitled to financial support when his/her needs are not met in any other way. The aim of the present study was to analyse the prevalence and impact of various illness factors and symptoms in social security recipients compared to non-recipients in a welfare state, in this case Sweden.

**Methods:**

A simple random sample of 20 100 individuals was selected from a national survey that covered all individuals in the 18–84 year age group in Sweden. A postal survey was thereafter conducted. Multiple logistic regression was employed as a statistical test. Odds ratio (OR) and a 95% confidence interval (CI) was used.

**Results:**

Social security recipients were found to have a significantly higher risk in most of the studied variables. Reduced psychological wellbeing measured by means of the GHQ12 was significantly higher in this group compared to the rest of the population (OR 1.41 CI 1.03–1.94) and their lack of trust was greater (OR 1.96, CI 1.45–2.66). They reported more sleep disturbances (OR 2.16, CI 1.58–2.94) and suffered from anxiety (OR 1.74, CI 1.28–2.36). Their dental health was worse (OR 2.44, CI 1.82–3.28) and they had more pain in their hands and legs (OR 1.57, CI 1.16–2.12). Social security recipients were more often humiliated (OR 1.79, CI 1.31–2.44) and exposed to threat (OR 1.69, CI 1.09–2.61). They were less physically active (OR 1.56, CI 1.17–2.08), had a poorer diet (OR 1.95, CI 1.45–2.63) and were more often smokers (OR 3.20, CI 2.37–4.33).

**Implication:**

The challenge for the welfare state consists of recognising the significance of both structural and lifestyle factors as a means of reducing the health gap.

## Background

Several recent social epidemiological studies have revealed inequalities in health around the world [1,2]. It has been established that belonging to a lower social stratum is, among other things, associated with poorer health as well as a higher risk of illness and early death from the most common diseases [3,4]. *Sweden *is a welfare state with a high standard of living, long standing prosperity and a relatively equitable distribution of national resources among the population [5]. One indicator of good health is that today, life expectancy in Sweden is one of the highest in the world [6,7]. The availability of high-tech health care, a high level of education and knowledge in addition to a wide range of activities organised by non-profit associations have contributed to high expectations in terms of health among Swedish citizens.

In Sweden social security is a means-tested financial allowance. The Social Services Act states that an individual is entitled to financial support when his/her needs are not met in any other way. From its inception, the Act was intended to deter individuals from seeking assistance, as it was considered shameful to receive help [8]. In 2004, the total number of people on social security was 417 491 (4.6%). The number of long-term social security recipients, i.e. those in receipt of social security payments for at least ten months, was 137 670, which corresponds to 1.5% of the total population. This represents an increase of approximately 50% between 1991 and 2003 [9,10].

A high proportion of social security recipients have a foreign background, although among younger ones the opposite applies. Women are in a clear majority among the older recipients, while men are more numerous in the middle age group. The likelihood of being a social security recipient is greater if one is unemployed [11], an immigrant, especially a refugee [12], a single mother or young and without educational qualifications [13].

The most vulnerable socio-economic groups have not obtained any share of the improved prosperity [14]. Social security recipients are also exposed to violation and threat as well as ridicule and humiliation to a significantly greater extent compared to those with a secure and permanent job [8] in addition to being at higher risk of illness [15]. Long-term social security recipients generally have poorer mental and physical health and are more likely to have substance abuse problems [16]. The health status of such vulnerable individuals has previously been studied from various perspectives. The majority of these studies were carried out in societies with extensive health gaps as well as cultural differences. Large-scale national socio-epidemiological studies of vulnerable groups in welfare societies have only been conducted to a lesser extent. Therefore, the aim of this study was to analyse the prevalence and impact of psychological and physiological ill-health and symptoms, psychological exposure and lifestyle habits among social security recipients compared to non-recipients in a welfare society.

## Methods

### Design

Sweden was chosen for the comparison between social security recipients and non-recipients. The study was carried out in 2004 and consisted of a national survey, the aim of which was to chart health conditions [17]. The survey was performed by Statistics Sweden (SCB) on the initiative of the National Institute of Public Health (FHI) [18].

### Population and sampling

The study population comprised all inhabitants of Sweden between the ages of 18 and 84.

A sampling framework was created that delimited, identified and allowed the variables to be linked to the objectives of the study. The sampling framework was based on the total population register (RTB), which has been administered by SCB since 1968 [19]. The register is an extract from the National Population Registers held by the Inland Revenue. The RTB is above all used as a basis for the preparation of statistics related to the size and composition of the population, distributed according to sex, age, civil status etc in the provinces and municipalities [19]. The number of individuals in this partition was 6 891 560, from which a Simple Random Sample of 20 100 subjects was drawn. A total of 12 166 individuals returned the questionnaire, corresponding to a response rate of 61%, of whom 267 were social security recipients (2.2%).

### Instrument

The questionnaire comprised 143 questions and was sent to the selected individuals by post. An attached information letter requested them to answer the questions and return the completed form to SCB. Three reminders were sent to those who failed to return the questionnaire at intervals of ten days. The present study only analysed the items pertaining to background variables, psychological and physiological ill-health, symptoms, psychological exposure and lifestyle habits.

### Psychological ill-health and symptoms

Reduced psychological wellbeing was assessed by means of the General Health Questionnaire (GHQ12), which has been developed as a screening instrument for mental illness [20]. The instrument, which has been validated and used all over the world, comprises 12 questions on an ordinal scale [21]. In the present study, the overall reliability of the instrument calculated by Cronbach's alpha, based on summation of all the items, was 0.90 (Mean = 22.03, SD = 5.2). Lack of trust was assessed by the question Do you believe that, in general, one can rely on most people? The question had a yes/no response alternative. The questions dealing with psychological symptoms were formulated as follows: Do you suffer from one or more of the following problems or symptoms? The response alternatives were "No", "Yes, mild problems", "Yes, serious problems". All of the above questions were dichotomised into dummy variables, where higher values indicated poorer health.

### Physiological ill-health and symptoms

Dental ill-health was measured by the following question, "How is your dental health"? The response alternatives ranged from very good to very bad. The question was weighted with reference to two other sub questions on dental health. In the same way as the questions dealing with psychological symptoms, the physiological symptoms were assessed by means of: Do you suffer from one or more of the following problems or symptoms? The response alternatives were "No", "Yes, mild problems" and "Yes, serious problems". All of these questions were dichotomised into dummy variables, where higher values indicated poorer health.

### Psychological exposure

Humiliation was assessed by means of a question about whether the respondent had been treated in such a way that he/she experienced humiliation during the past three months. The response alternatives consisted of "No", "Yes, once" and "Yes, several times". This question was dichotomised into "Yes and No". Exposure to threat was measured by means of the following question: Have you been exposed to threat or threats of violence during the past twelve months that made you feel afraid"? The response alternatives were "Yes and No".

### Lifestyle habits

Physical inactivity was measured by a question on an ordinal scale, from regular physical exercise to sedentary leisure hours. Questions about poor diet were measured by three items, which ranged from daily consumption of fruit to the use of cooking fat on bread. The three questions were weighted and thereafter dichotomised to one variable. Daily smoking was measured by the question "Are you a daily smoker"? The answer was "No or Yes". Risky alcohol consumption was measured by two questions, "How much?" and "How often?" do you consume alcohol. The answers ranged from "Four times a week or more" to "Never", and from "Daily consumption" to "Never", respectively. These two variables were amalgamated into one dichotomised variable with regard to weighting. The internal response frequency was between 85.6 and 99%. Age, height and weight were chosen as continuous variables, while the remaining information about citizenship was drawn from the national register. In addition to the variables from the questionnaire, several were derived from the RTB, the education register as well as the Register of Income and Wealth (IOF) [22].

### Data collection and processing

The questionnaire was distributed by post with three reminders. SCB processed the raw data, after which the responses were complemented by register variables from the RTB as well as weighted for the purpose of adjustment to population level. The analysis of the material was based on existing health indices prepared by the FHI in collaboration with a national expert group. The overall study material was standardized in terms of age. A minor adjustment was made whereby some questions were amalgamated in order to facilitate in-depth analyses.

### Ethical considerations

Permission for the study and the confidentiality test were dealt with by SCB in collaboration with the FHI, after which an internal agreement was reached between them with regard to the confidentiality test to be carried out at the time the variables from the RTB, education and income tax registers were to be handed out. In addition, a secrecy agreement was drawn up between FHI and SCB, which set out the way in which the de-anonymised data material and questionnaires were to be handled. The legal section of SCB was notified about the treatment of personal data in the study. An information letter was enclosed with the questionnaire and provided details about the background, aims and design of the study together with details about the handling of data, the fact that participation was voluntary and that the responses would be treated confidentially. The linkage to personal details was removed three months after the delivery of data was completed.

### Calculation of weights

Weights were calculated by means of calibration in order to reduce skewness in terms of drop out and allow the result to be adjusted to population level. The purpose of upward adjustment is to obtain a result that is representative of the population as a whole. Weighting compensates for dropout but not partial dropout, which means that the numbers stated in the tables may differ. *Calibration weighting *was employed in order to compensate for the skewness in response frequency between the sub groups. The weighting was based on the assumption that the sample was a satisfactory representation of the population, which means that over and under cover are negligible. Thus, the weights obtained could be used in the calculation of statistical measures of a descriptive nature.

### Statistical analysis

Multiple logistic regression was used as a statistical test [23] and separate analyses were performed for all categories of variables. The background variables of age, sex and nationality were taken into account. The analysis was carried out in two steps. In the first, a test was performed on each of the variables, including the background variables. In the second, all the significant variables from step one were analysed together. A double-sided test with an odds ratio (OR) and a 95% confidence interval was used.

## Results

Social security recipients were found to have significantly poorer health than non-recipients in most of the studied variables (Table 1). The majority reported lack of trust, sleep disturbances, tiredness, anxiety, dental ill-health, bodily pain, exposure to humiliation and physical inactivity. The results of the multivariate analysis revealed significantly lower scores for social security recipients in most of the variables analysed in the study, with the exception of high-risk alcohol consumption (Table 1). Ill-health as measured by means of the GHQ 12 was significantly higher in this group compared to non-recipients (OR 1.41, CI 1.03–1.94). They experienced a greater lack of trust (OR 1.96, CI 1.45–2.66) as well as more sleep disturbances (OR 2.16, CI 1.58–2.94) and anxiety (OR 1.74, CI 1.28–2.36). They also exhibited more dental ill-health (OR 2.44, CI 1.82–3.28). All physiological symptoms were significantly more common among social security recipients compared to non-recipients in step I of the analysis, while hand/arm/leg or knee pain was the most frequent symptom when all the variables were taken into account (OR 1.57, CI 1.16–2.12). They had more often been humiliated (OR 1.79, CI 1.31–2.44) and had been exposed to threat to a greater extent (OR 1.69, CI 1.09–2.61). The social welfare recipients were less physically active (OR 1.56, CI 1.17–2.08), had a poorer diet (OR 1.95, CI 1.45–2.63) and were significantly more likely to be regular smokers (OR 3.20, CI 2.37–4.33) (Table 1). These variables were the only significant indicators when all of the variables were combined in the multivariate analysis (Table 1).

**Figure 1:**
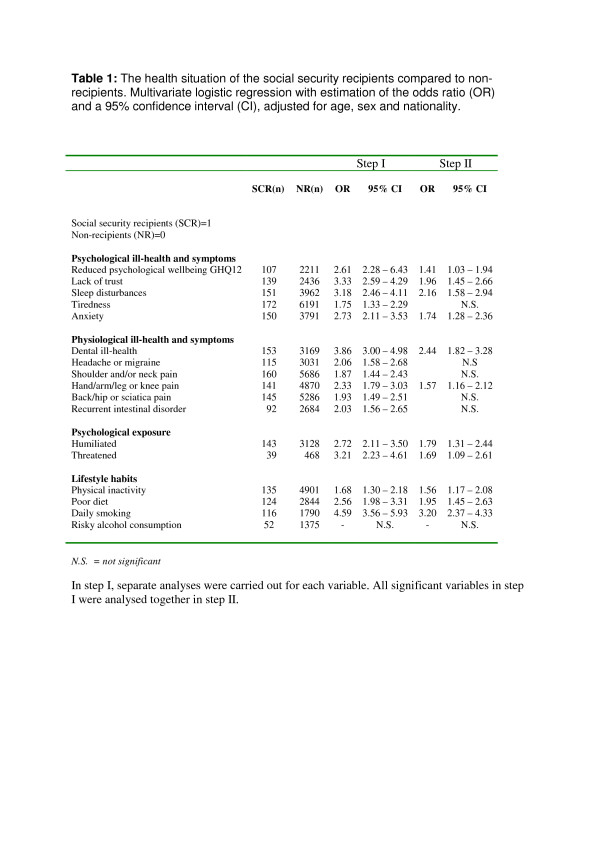
**Table 1: The health situation of the social security recipients compared to nonrecipients.** Multivariate logistic regression with estimation of the odds ratio (OR) and a 95% confidence interval (CI), adjusted for age, sex and nationality.

## Discussion

### Method issues

SCB's quality declaration pertaining to official statistics was used in order to ensure high quality and cover all quality dimensions [24]. Of the study population, 2.2% were social security recipients, which is a lower percentage than the official figure. A possible explanation may be that social security recipients are less willing to take part in studies and that the official statistics are based on the total population, while the present study employed an age interval of 18–84 years. The updated RTB register was used in the study in order to reduce cover errors. It was revealed that 96 individuals constituted over cover. These subjects had either died or moved abroad. No under cover was observed. The questionnaire was scrutinised by the Measurements Department of Statistics Sweden in order to minimise measurement error and carry out a check of, among other things, permissible values. The measurement errors related to the RTB register variables were deemed to be small. Dropout errors were carefully scrutinised and corrected by means of the standardisation and calibration of the variables analysed in the study.

### Results discussion

The present analysis, which is built on comprehensive and up-to-date Swedish data, reveals significant differences between the study groups. The social security recipients were found to have worse health values in most of the study variables compared to non-recipients. The results of this study demonstrate that lack of trust at interpersonal level is considerably higher among social security recipients compared to non-social security recipients. Lack of trust is highest in housing estates with a large income gap, weak social relationships and where violence is commonplace [25]. Trust in other people is important as well as a key component in the life of each individual. It is developed at an early stage and strengthened by a secure environment in the formative years [26]. At interindividual level, trust has been found to be related to, for example, self-rated health, satisfaction with life, functional health and mortality [27].

Social security recipients had experienced humiliation and threat to a greater extent than non-recipients. Several studies carried out in Scandinavia have revealed that many people associate living on social welfare with shame [28,29]. It has been suggested that shame (e.g. humiliation, insult and ridicule) is an important factor behind violence [30,31]. Humiliation may lead to a vicious circle of more threats and violence and it is well known that insecurity and exposure to violence are more common in unequal societies [25]. Furthermore, humiliation can be an important contributory factor in reduced psychological wellbeing [32]. Findings in theses studies demonstrate that social security recipients have reduced psychological wellbeing (GHQ12) compared to non-recipients, which is similar to the results from other studies [10]. Social security recipients' constant worry about their poor financial situation can lead to sleep disturbances and anxiety, which factor may also explain their reduced psychological wellbeing.

The present study revealed the unhealthy lifestyle of the social security recipients, comprising a greater lack of physical activity and poorer diet as well as more often being habitual cigarette smokers. The highest risk factor in the multivariate analysis was daily smoking, which, in combination with unhealthy food and lack of physical activity, should be considered a risk over time. Existing research has revealed that an unhealthy lifestyle, such as smoking and poor eating habits, is more widespread among vulnerable socio-economic groups [15,33], and that these groups are more frequently exposed to several risk factors [34]. In healthy subjects it has been found that the more risk factors they exhibit such as smoking, physical inactivity, poor diet and high-risk alcohol consumption, the higher the mortality risk when adjusted for age, sex, BMI and socioeconomic group [35]. Social security recipients have higher levels of unhealthy lifestyle habits, which, according to previous studies, only appeared to explain a third of their higher mortality [36]. Other studies have demonstrated that social security recipients exhibit more high-risk alcohol consumption than non-recipients [16]. While the present study revealed a similar tendency, it was not significant. Possible explanations may be that the present study includes a large proportion of social security recipients with an immigrant background whose religion forbids alcohol. Social security recipients have poorer dental health than non-recipients. This can be due to worse dental hygiene, unhealthy lifestyle habits, reduced psychological well-being as well as a poor financial situation. From a structurally oriented perspective, structural conditions probably play a major role in the worse health situation of this group. Social security recipients are often unemployed and dependent on social benefits in order to support themselves. The problem of being unable to support oneself often goes hand in hand with worse living conditions, a lifestyle that is damaging to health in combination with poor physical and mental health [15].

In recent decades the socio-economic gaps have increased in Sweden as well as in several other western European countries [37]. A large difference in health is characteristic of societies that have major social gaps. Being marginalised and having low status in a welfare society in terms of finances, education, living conditions and culture often leads to reduced autonomy and thus less opportunity to influence one's situation. According to Marmot, being marginalised and reduced to the bottom rung of the welfare society's socio-economic ladder has in itself a negative effect on health [36]. His theory holds that health is not absolute but relative. Accordingly, an individual's economic situation is not the most important factor for health, but rather his/her standard of living in relation to the people in his/her environment. This theory is supported by the fact that society today is made up of social hierarchies and that health is worse the lower in the hierarchy one finds oneself [36]. The consequence of this theory is that the inequality in terms of health cannot be levelled out by financial allowances to the poorest as long as the richest become richer, thereby increasing the socio-economic gap.

Finally the study shows clear differences in psychological and physiological ill-health and symptoms, psychological exposure and lifestyle habits between social security recipients and non-recipients. These differences indicate a health gap between the two groups. There is a great deal of evidence to support an association between, on the one hand, psychological and physiological ill-health, symptoms, psychological exposure and risk factors and, on the other, exposure to structurally related risk factors, for example unemployment, financial worries, poverty etc. In the work to increase health and well-being it is therefore vital to reduce powerlessness and promote autonomy by providing opportunities for each individual to satisfy his/her own needs, solve his/her own problems and have control over his/her own life. This, together with the conclusions of Marmot's theory, constitutes a major challenge for the welfare society in its work to reduce differences in health status.

## Competing interests

The authors declare that they have no competing interests.

## Authors' contributions

AB contributed to the conception of the manuscript and participated in the survey process in addition to designing the study, collecting data, performing the analysis and writing the article. E-CL contributed to the study design, conception and critical revision of the manuscript, as well as providing complementary views. BS critically revised the manuscript, provided additional comments and contributed to the final draft. HB contributed to the study design, conception and critical revision of the manuscript, as well as providing complementary views. AB, E-CL, BS and HB wrote and approved the final manuscript.
